# Complementary Strategies to Unlock Biosynthesis Gene Clusters Encoding Secondary Metabolites in the Filamentous Fungus *Podospora anserina*

**DOI:** 10.3390/jof9010009

**Published:** 2022-12-21

**Authors:** Ling Shen, Catherine Roullier, François-Hugues Porée, Thomas Gaslonde, Ludivine Riffault-Valois, Olivier Grovel, Gwenaël Ruprich-Robert, Florence Chapeland-Leclerc

**Affiliations:** 1 School of Life Science, Jiangsu Normal University, Xuzhou 221116, China; 2 Laboratoire Interdisciplinaire des Energies de Demain (LIED), UMR 8236, CNRS, Université Paris Cité, F-75013 Paris, France; 3 Institut des Substances et Organismes de la Mer (ISOMER), UR 2160, Nantes Université, F-44000 Nantes, France; 4 Laboratoire de Chimie Thérapeutique, UMR 6226, ISCR, CNRS, Faculté de Pharmacie, Université de Rennes 1, F-35065 Rennes, France; 5 Cibles Thérapeutiques et Conception de Médicaments (CiTCoM), UMR 8038, CNRS, Faculté de Pharmacie de Paris, Université Paris Cité, F-75006 Paris, France; 6 Institut Pluridisciplinaire Hubert Curien, UMR 7178, CNRS, Université de Strasbourg, F-67000 Strasbourg, France

**Keywords:** *Podospora anserina*, secondary metabolites, complementary strategies, metabolomics

## Abstract

The coprophilous ascomycete *Podospora anserina* is known to have a high potential to synthesize a wide array of secondary metabolites (SMs). However, to date, the characterization of SMs in this species, as in other filamentous fungal species, is far less than expected by the functional prediction through genome mining, likely due to the inactivity of most SMs biosynthesis gene clusters (BGCs) under standard conditions. In this work, our main objective was to compare the global strategies usually used to deregulate SM gene clusters in *P. anserina*, including the variation of culture conditions and the modification of the chromatin state either by genetic manipulation or by chemical treatment, and to show the complementarity of the approaches between them. In this way, we showed that the metabolomics-driven comparative analysis unveils the unexpected diversity of metabolic changes in *P. anserina* and that the integrated strategies have a mutual complementary effect on the expression of the fungal metabolome. Then, our results demonstrate that metabolite production is significantly influenced by varied cultivation states and epigenetic modifications. We believe that the strategy described in this study will facilitate the discovery of fungal metabolites of interest and will improve the ability to prioritize the production of specific fungal SMs with an optimized treatment.

## 1. Introduction

Fungi possess huge potential to produce a wide range of secondary metabolites (SMs) including both beneficial and/or detrimental small molecules [1,2]. Genes responsible for the biosynthesis of these SMs are typically arranged in a multigene biosynthetic gene cluster (BGC) throughout the genome [3]. However, to date, only a limited number of SM pathways have been elucidated in fungi because the vast majority of fungal BGCs are frequently cryptic or expressed at a low level under standard laboratory growth conditions [4]. Therefore, as an already validated source of useful bioactive natural products, the actual capabilities of fungi that produce far fewer SMs than expected are substantially underestimated [5]. In this context, strategies for activation of the silent BGCs are prerequisites to unearthing the hidden fungal SMs.

Several targeted strategies have been developed to awaken silent fungal BGCs, which mainly include (i) genetic manipulation of a cluster-specific regulator, (ii) promoter exchange, and (iii) heterologous expression [6]. The gene encoding a cluster-specific transcription factor (TF) can be located within a BGC and regulates the expression of all corresponding clustered genes together [7]. Hence, the overexpression of the pathway-specific transcriptional activator and the inactivation of the repressor are both undoubtedly considered targeted approaches that lead to the activation of silent BGCs and the identification of novel SMs in many fungal species. This strategy was exemplified by aspyridone in *Aspergillus nidulans* [8] or trichosetin in *Fusarium fujikuroi* [9]. Given that many BGCs are not under the control of identified TF and that a cluster-specific TF in some cases is not available, an alternative strategy consists of the replacement of the endogenous promoters of all genes within a BGC with constitutive or readily inducible promoters [4,10]. This approach was successfully applied in *A. nidulans*, for instance, where native promoters of all genes of several cryptic BGCs were sequentially replaced with an inducible alcohol dehydrogenase (*alcA*) promoter, which finally triggers the synthesis of fellutamide B [11,12], polyene aspernidgulene A1 [13], conidiophore pigments [14], and microperfuranone [15]. For fungal organisms, which are uncultivable or not genetically tractable under laboratory conditions, the heterologous expression strategy is suitably applied to transfer and express the desired BGC in a surrogate host, such as genetically engineered in *Escherichia coli*, *Saccharomyces cerevisiae,* and *Aspergillus* spp. [16,17,18]. The metabolome of heterologous hosts is commonly well-characterized, and some of them present an especially limited SM production profile and therefore low chemical background noise that simplify the identification of SMs of the corresponding appended BGCs. Harvey et al. [19] developed an optimized Hex platform in *S. cerevisiae* for the rapid examination of 41 cryptic BGCs from diverse fungal species. The expression of plenty of silent BGCs and the discovery of a wide range of fungal SMs, e.g., asperfuranone or neosartoricin B, also benefit from the efficient heterologous expression system [20,21]. Briefly, a strong link between candidate BGCs and corresponding natural products can be therefore established owing to the targeted strategies mentioned above.

Compared with targeted strategies, global approaches offer a higher throughput of SMs production, such as (i) global regulator manipulation, (ii) epigenetic modification, and (iii) variation of growth conditions [2,22]. The most well-studied global regulator with regard to fungal SMs is *LaeA*, in which deletion and overexpression alter the expression of BGCs and contribute to the discovery of novel natural products from microbial sources [23,24,25,26,27]. In contrast to *LaeA*, the negative global regulator *mcrA* was also identified and is involved in the downregulation of a large number of SMs-related genes in *Aspergillus* and *Penicillium* species [28]. However, the manipulation of transcriptional regulators does not always induce the expression of silent BGCs, which is in part due to epigenetic regulation [29]. Chromatin in a repressed state can be modulated into an activated state by remodeling the chromatin structure, which thereby triggers the activation of cryptic BGCs. Accordingly, two methods aimed at the alteration of fungal epigenome were developed: Molecular manipulation of histone-modifying enzymes and the treatment of fungal culture with chemical agents, which interact with these enzymes. These methods are widely used to stimulate novel metabolite production. Chromatin regulation of fungal metabolism by mutagenesis of genes encoding histone methyltransferase, acetyltransferase, demethylases, and deacetylases indeed enabled the pleiotropic activation of silent metabolites in numerous fungi [30,31,32,33]. Meanwhile, the addition of DNA methyltransferase or histone deacetylase small-molecule inhibitors such as 5-azacytidine, RT-108, vorinostat (also known as suberoylanilide hydroxamic acid, SAHA), sodium valproate (VS), octanoylhydroxamic acid (OHA), or trichostatin A, has also been proven to be fruitful in inducing cryptic SM production [34,35,36,37]. In addition, an alternative that is sometimes easier to implement is to modify the fungal growth conditions that mimic their natural environment, in order to trigger the expression of some cryptic BGCs. Both abiotic modifications by modulating the physicochemical variables of culture conditions and biotic interspecies cross-talk by co-culturing of fungi with other microorganisms have been proven to efficiently influence BGC expression and activate secondary metabolites’ output [38,39,40,41,42]. Nevertheless, such untargeted approaches, also known as the “one strain many compounds” (OSMAC) approach, often simultaneously induce multiple fungal BGCs and lead to the production of a complex mixture of SMs, which complicates the prediction of diverse outcomes [43]. As a result, untargeted approaches are intrinsically empirical and commonly integrated as complements to other targeted strategies. However, to our knowledge, fewer data about the comprehensive assessment among different global approaches that trigger cryptic SMs production are yet available, and the methodology evaluation of each untargeted approach to broaden the SM spectrum of fungi is still poorly achieved.

*Podospora anserina*, frequently recovered from herbivore dung, is a typical coprophilous ascomycete fungus [44]. Dung-inhabiting fungi are predominately known to produce various SMs that act as chemical weapons to enhance ecological fitness [45,46]. Indeed, genome mining of *P. anserina* revealed the presence of a large number of putative BGCs, including 18 PKS, 8 NRPS, and 3 hybrid PKS/NRPS [47].

However, to date, the metabolome diversity of this fungi is underexplored. A few sets of secondary metabolites have been isolated including anserinones A and B, two benzoquinones exhibiting antifungal, antibacterial, and cytotoxic activities [48], and sterigmatocystin (ST), a polyketide demonstrating larvicidal and antifungal activities [49,50,51]. To the best of our knowledge, apart from the ST gene cluster that has been experimentally confirmed and functionally characterized in *P. anserina*, only seven putative BGCs displaying lower sequence similarity might be assigned to known molecules, while none of the other clusters can be correlated to natural products [44,50]. Genomics-driven BGC detection and several chemical investigations have been conducted so far, implying that *P. anserina* is a prolific yet unexploited natural product source.

The purpose of this work is to study and compare the metabolome chemodiversity of *P. anserina* following three strategies, in order to demonstrate their mutual complementarity: (1) Variation of culture conditions with solid and liquid culture media, (2) chromatin remodeling through the addition of the chemical epigenetic stimulant SAHA in fungal cultures, and (3) chromatin modification via the genetic deletion of *Kmt6,* which encodes the histone H3K27 methyltransferase. The impact of these global approaches on the SMs’ production was analyzed via LC-HRMS of the fungal extracts. Metabolomics studies were carried out using both univariate and multivariate approaches. Altogether, these results evidence the positive effect of a global deregulation strategy to activate SM production but also the mutual complementarity to generate diversity.

## 2. Materials and Methods

### 2.1. Fungal Strains and Growth Conditions

*P. anserina* wild-type strain ‘S’ (big S) was the reference strain used for sequencing [47,52]. The *Kmt6* mutant strain, in which the gene encoding the histone H3K27 methyltransferase *Kmt6* was deleted, was kindly provided by Dr. F. Malagnac [53]. The standard medium used was M2 (KH_2_PO_4_ 0.25 g/L, K_2_HPO_4_ 0.3 g/L, MgSO_4_ 0.25 g/L, Urea 0.5 g/L, Thiamine 0.05 mg/L, Biotine 0.05 µg/L, Citric Acid 5 mg/L, ZnSO_4_ 5 mg/L, CuSO_4_ 0.25 mg/L, MnSO_4_ 50 µg/L, Boric Acid 50 µg/L, Natrium Molybdate 50 µg/L, Iron Alum 1 mg/L, Dextrin 5.5 g/L) with pH maintenance at 7 by a phosphate buffer. All the protocols including standard culture conditions and genetic manipulation for this microorganism are described in [44] and can be accessed at http://podospora.i2bc.paris-saclay.fr (accessed on 1 February 2020).

All the experiments were performed in quadruplicates in 24-well plates containing 2 mL of M2 minimal medium per well, and this medium can be made solid by adding 10 g/L of agar. Note that a total of eight replicates, divided between the two 24-well microplates required for the experiments, were made for the experiment involving the WT strain in the M2 liquid medium. Aliquots of 3 μL of the standardized fragmented mycelial suspension derived from a 1 cm^2^ section of fresh mycelia from the agar plug were respectively inoculated into each well. Based upon experiments previously conducted on the ST production in *P. anserina* [50], plates were then stationarily incubated at 27 °C in the dark for 8 days. For the addition of the epigenetic modifier, SAHA (Sigma-Aldrich, St. Louis, MO, USA), which dissolved in DMSO, was aseptically added before inoculation to a final concentration of 100 μM in the M2 liquid culture medium. The solid M2 medium, liquid M2 medium, and liquid M2 medium supplemented with 100 μM SAHA but without inoculum were set as blank controls to detect potential contaminations and monitor any metabolites of media components.

### 2.2. Fungal Culture Extraction

All whole fungal cultures (mycelia and media) were crushed by Fastprep and then treated with 3 mL of ethyl acetate (EtOAc) using ultrasonication for 30 min to obtain both intra- and extracellular metabolites. After the separation of the organic layers, the culture residues were macerated with 3 mL of EtOAc overnight. The combined organic phases were dried over MgSO_4_ and evaporated to dryness leading to an organic extract. Media without inoculated fungi were also extracted following the same protocol and considered controls. The samples were solubilized in methanol (MeOH) at the concentration of 0.5 mg/mL and then filtered through a 0.45 μm filter before analysis.

### 2.3. LC-MS/MS Analysis

The collected samples were analyzed with a UPLC-UV apparatus (Acquity, Waters) coupled with a Q-TOF MS system (microTof QII, Bruker) using an ESI source in positive ionization mode. All samples were separated on Accucore C18 column (100 × 3 mm, 2.6 μm particle size) equipped with a guard cartridge (10 × 3 mm, 2.6 µm) using 0.1% formic acid-deionized water (A) and acetonitrile (B) as solvents. The optimized gradient elution program was as follows: 0–1 min, 3% B; 1–15 min, 3–100% B; 15–17 min, 100% B; 17–18 min, 100–3% B; 18–23 min, 3% B. The injection volume was 5 μL and the flow rate was 0.4 mL/min. The wavelength was set at 254 nm. Samples (5 μL) at the concentration of 0.5 mg/mL in MeOH were injected. MeOH blanks were injected randomly during the analysis sequence. A mixture of all extracts at the same concentration was also prepared as a quality control and regularly injected throughout the sequence. MS/MS analysis was performed using a capillary voltage of 4.5 kV. Drying and nebulization gas (nitrogen) flow rates were set at 10.5 L/min and 59.5 psi, respectively, with a source temperature of 200 °C. The *m*/*z* range recorded was 100–1500. MS/MS analyses were performed using the AutoMS/MS in Data Dependent Analysis mode. Two precursor ions with a minimum intensity of 4000 were selected per fragmentation cycle. Four consecutive spectra were allowed before exclusion and released after 0.1 min. The total cycle duration was 1.5 s. The collision energy was set at 30 eV for mono-charged ions of 100 *m*/*z* and 45 eV for 1000 *m*/*z*.

### 2.4. Data Processing

UPLC-HRMS/MS profiles were converted into *.mzXML format using MS convert with MS levels 1–2 [54]. The profiles were submitted to peak picking using MZmine 2 [55]. The first step of mass detection was performed with a noise level set at 150. The ADAP chromatogram builder algorithm was then used with the following parameters: Minimum group size of 4 scans, a group intensity threshold of 300, the minimum highest intensity of 100, and *m*/*z* tolerance of 0.02. Chromatogram deconvolution for each *m*/*z* was further carried out with the ADAP wavelet algorithm applying the following parameters: s/n threshold at 10, minimum feature height set at 500, coefficient/area threshold set at 25, peak duration range of 0–5 min, and *t*_R_ wavelet range of 0.04–1.50 min. Then isotope removal was performed with the isotopic peak grouper with an *m*/*z* tolerance of 0.01, a *t*_R_ tolerance of 0.05 min, and a maximum charge of 3. The most intense isotope was chosen as the most representative one. Alignment was carried out with the join aligner algorithm allowing 0.02 *m*/*z* tolerance (weight: 1) and 0.15 min for *t*_R_ tolerance (weight:1). Gap filling was then performed using the same parameters for *m*/*z* and *t*_R_ tolerance. Duplicates were removed with 0.02 *m*/*z* tolerance and 0.1 min *t*_R_ tolerance, and the «new average» method was used for intensities and areas to be kept. Another step of filtration was conducted by removing all peaks present in blank and medium samples (negative controls). Then, a total of 1756 features (*m*/*z*_t_R_) were obtained.

All ions reported by previous studies as corresponding to SAHA features were searched on the raw data and checked through MS spectra [56,57]. Six retention times were then identified (*t*_R_ 5.96/6.20/6.30/7.41/9.10/9.63 min) as characteristic of compounds originating from SAHA. In the data matrix previously obtained, all the features that had the same *t*_R_ (±0.1 min) or a similar peak area profile among the samples (Pearson ≥ 0.8), such as feature 265.155 at 5.96 min, were removed. In total, 203 features were then removed leading to a matrix with 1553 features for further statistical analyses.

### 2.5. Statistical Analysis

The data matrix was analyzed by using both the open-source software Rstudio version 1.4.1103 (© 2022–2021 RStudio, PBC, Boston, MA, USA) and the MetaboAnalyst 4.0 website (http://www.metaboanalyst.ca, accessed on 12 February 2020) to identify unique and common compounds to all media using Venn diagrams and univariate analyses. After uploading, the dataset containing 1553 features was normalized by sum and Pareto scaling. One-way ANOVA was performed using a *p*-value < 0.001 and Fisher’s LSD as post-hoc analysis, which determined the top 671 features with the most significant variable mean for peak areas among the media. Hierarchical Clustering Analysis (HCA) of these 671 features using a Heatmap was set using the Euclidean distance measure and the “Ward” algorithm for clustering. Multivariate analyses were further conducted by submitting the normalized data matrix to principal component analysis (PCA) and Partial Least Square discriminant analysis (PLS-DA).

Heatmaps, PCA score plots, and loadings plots were obtained from the MetaboAnalyst 4.0 website. Other plots were obtained using RStudio. The tables generated by MetaboAnalyst for ANOVA, PCA, and PLS-DA analyses were downloaded, combined, and analyzed to highlight features of interest.

### 2.6. Dereplication

Among features that were highlighted by previous statistical analyses, different adducts and fragments were identified, allowing the detection of the protonated molecule for most of them. Molecular formula prediction based on the [M+H]^+^ and MS-spectra-deduced adduct exact mass and isotopic patterns was performed using ChemCalc online software (https://www.chemcalc.org, accessed on 12 February 2020). Formulae obtained with an *m*/*z* error lower than 20 ppm for the deduced monoisotopic mass were filtered using the Kind and Fiehn’s Seven Golden Rules [58] and then searched in the Dictionary of Natural Products (DNP), limiting the queries to natural products isolated from ascomycetes (DNP 30:1 copyright © 2022). In addition, compounds already isolated from *Podospora* genus were retrieved by searching the data matrix for all ions corresponding to either [M+H]^+^, [M+Na]^+^, [M+K]^+^, [2M+H]^+^, or [2M+Na]^+^ with an *m*/*z* tolerance of 0.01.

## 3. Results

### 3.1. Metabolomic Study of P. anserina in Different Conditions

**Figure 1:**
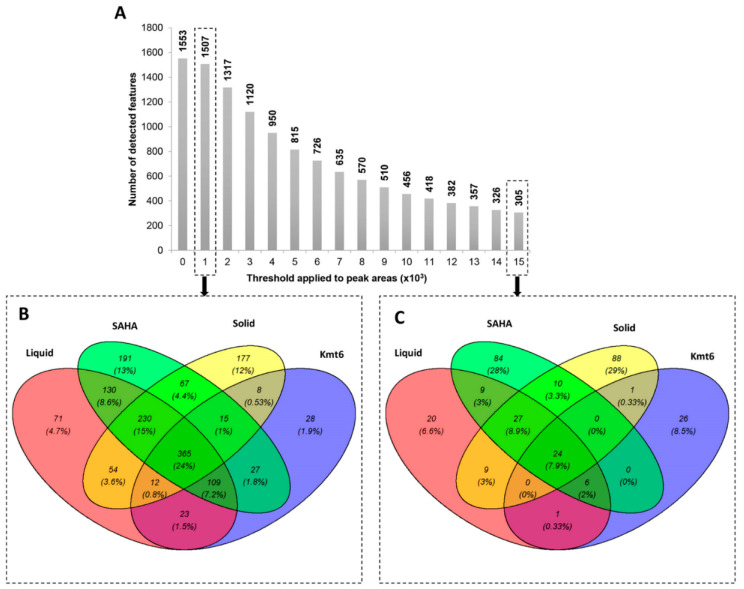
Representation of overlapping features from the different conditions tested for SM production in *P. anserina* after 8 days of culture. (**A**) Variation of the number of features considered with the threshold on peak areas applied with (**B**,**C**) corresponding to the Venn diagrams obtained with the intensity thresholds set to 1000 and 15,000 a.u., respectively. “Liquid”: M2 liquid medium inoculated with WT; “Solid”: M2 solid medium inoculated with WT; “SAHA”: M2 liquid medium supplemented with 100 μM SAHA and inoculated with WT; “Kmt6”: M2 liquid medium inoculated with the *Kmt6* mutant strain. All the experiments were performed in quadruplicates, except for the condition “Liquid” for which 8 replicates were performed.

The *P. anserina* wild-type strain (WT) was cultivated on a liquid or solid M2 culture medium, in order to compare the SMs produced under two different conventional culture conditions. Additionally, in order to investigate the impact of a chromatin modification on the SM production in *P. anserina*, (i) the epigenetic regulator SAHA was added to the liquid M2 medium inoculated with WT and (ii) the *Kmt6* mutant strain missing the histone H3K27 methyltransferase was cultivated under the same liquid M2 conditions. After 8 days of cultivation, extraction of the whole fungal cultures was conducted, and each sample was analyzed by LC-HRMS. Then, the first comprehensive data comparison, obtained from the four conditions tested (WT on a solid medium, WT on a liquid medium, WT on a liquid medium + SAHA, and Δ*Kmt6* on a liquid medium), revealed interesting outcomes (Figure 1). With a minimal threshold (intensity of 1000 a.u.) allowing background and minor peaks removal, out of the 1507 remaining features, only 365 (24%) were found to be present in all conditions, meaning that the global molecular content of the strain clearly varied from one condition to another. A unique feature refers to data only detected in one of the conditions tested according to the threshold applied. Interestingly, the addition of SAHA in the medium, as well as cultivation of the strain on the solid medium, allowed the detection of the most important number of unique features, with 191 (13%) and 177 (12%) unique features highlighted, respectively. Contrarily, the mutant strain *ΔKmt6* was less productive with only 28 (1.9%) unique features. However, this number was still huge with a higher threshold (of 15,000) with 26 (8.5%) unique features, while all the other conditions had lower numbers. This could be explained by the fact that the mutant strain *ΔKmt6* was able to produce unique features in a more significant amount than the strains cultivated in other conditions. Interestingly, with a higher threshold (of 15,000), only 24 of the 305 detected features (7.9%) were found to be common to the four conditions. This means that the most abundant peaks found among the different samples mostly correspond to features overexpressed in only one or another condition (72% of the features detected). This result highlights the fact that, individually, all the different conditions tested were able to modulate the metabolism with specific profiles each time.

**Figure 2:**
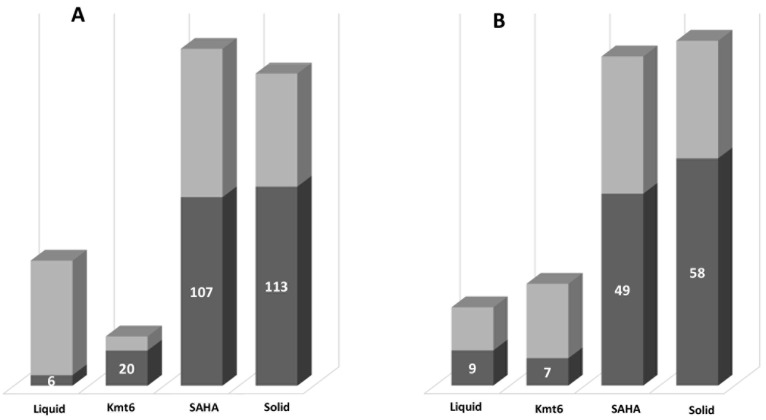
Number of “unique” features with significantly different means (in dark grey) among all previously detected as “unique” for each condition tested for SM production on *P. anserina* after 8 days of culture, according to a threshold of 1000 (**A**) and 15,000 (**B**) and a *p* value < 0.001 on ANOVA test. “Liquid”: M2 liquid medium inoculated with WT; “Solid”: M2 solid medium inoculated with WT; “SAHA”: M2 liquid medium supplemented with 100 μM SAHA and inoculated with WT; Kmt6: M2 liquid medium inoculated with the *Kmt6* mutant strain. All the experiments were performed in quadruplicates, except for the condition “Liquid” for which 8 replicates were performed.

An analysis of variance (ANOVA) was then performed to compare the differences in metabolite production between the four groups of conditions. Features with significantly different means (*p* value < 0.001) were then highlighted among the previously detected “unique” features (in dark grey in Figure 2). It appeared that the highest proportion of significant unique features was observed on the solid medium, regardless of the threshold used. In fact, among all features of the matrix, 671 of them presented a *p* value < 0.001 in the ANOVA analysis, with 113 features (with peak areas above 1000) corresponding to features overexpressed in the solid medium. Referring to the Kmt6 mutant, we showed that among the 26 features labelled as unique in this condition with a 15,000 threshold, only 7 were actually significant. This is why we refined this analysis to better point out features of interest. Interestingly, for the features detected in all four conditions, more than half presented significantly different peak areas (164 out of 365 or 14 out of 24), meaning that, even if produced in all conditions, these features are not expressed at the same level. The features present in all conditions but with a *p* value > 0.001 actually corresponded to features with relatively huge peak area dispersion among the samples.

**Figure 3:**
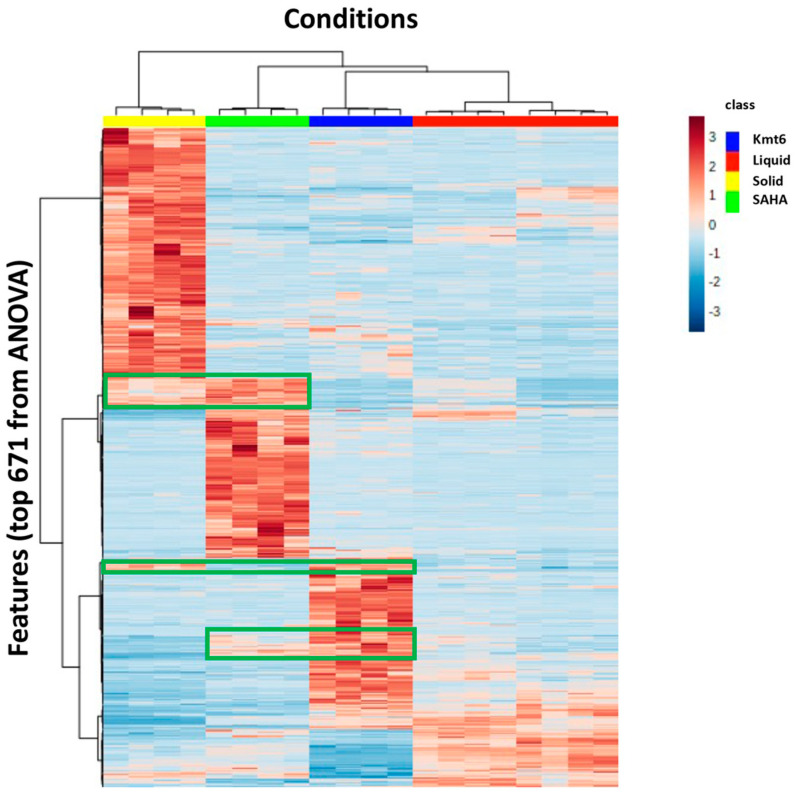
Hierarchical clustering analysis (HCA) of the most significant variable features (*p*-value < 0.001): 671 features (determined by their *m*/*z* ratio and retention time (*t*_R_)) among the samples corresponding to the four different conditions and represented on a heatmap, ranging from red color for high peak areas to blue. The heatmap was established by using the normalized and scaled peak areas of the 671 features. “Liquid”: M2 liquid medium inoculated with WT; “Solid”: M2 solid medium inoculated with WT; “SAHA”: M2 liquid medium supplemented with 100 μM SAHA and inoculated with WT; Kmt6: M2 liquid medium inoculated with the *Kmt6* mutant strain. All the experiments were performed in quadruplicates, except for the condition “Liquid” for which 8 replicates are presented. The green squares highlight some features that are detected in at least two different conditions.

A further investigation was performed by generating a heatmap presenting the 671 features with a *p* value < 0.001 in the ANOVA analysis (Figure 3) associated with the hierarchical clustering analysis (HCA) of the samples. It showed a clear distinction between the conditions investigated and the same four groups clearly appeared, highlighting their complementarity in terms of SM production. For some features, their occurrence was found to be similar in two conditions as exemplified in Figure 3 (green squares). It could be noted that the liquid culture condition has the lowest level of features detected, while the addition of SAHA led to a high increase in the number of SM features when compared to other conditions. To a lesser extent, the number of additional features is comparable in solid culture conditions and with the *ΔKmt6* strain with, again, very different expression profiles and few redundancies.

An unsupervised comparison of the chemical profiles was then performed by a principal component analysis (PCA) on the Pareto-scaled data matrix (Figure S1). Two clusters corresponding to the culture of the WT strain in a liquid medium were observed. They were explained by the two different plates they came from. However, they were still clustered and separate from the other conditions, allowing us to conduct further analyses on the score plot using the first two principal components. In fact, these latter explained most of the variability between the groups with 24.8% and 21.6% of the variance explained for PC1 and PC2, respectively. PC3 most corresponded to the differentiation between the two sets of the WT strain cultivated on a liquid medium on two different plates, namely, PC4 to an outlier of this same condition and PC5 to an outlier sample from the *Kmt6* mutant strain (Figure S2).

3D score plots using PC1, PC2, and PC3 highlighted four groups of samples indicating differences in SM production. For example, samples from the WT strain cultivated on the M2 medium in a liquid or solid state evidenced two separate groups according to the nature (liquid vs. solid) of the culture medium. These data illustrate the dramatic influence of the growth medium on SM production regardless of the source of nutriments. This separation between metabolic profiles from the four different conditions (meaning WT on the solid medium, WT on the liquid medium, WT on the liquid medium + SAHA, and Δ*Kmt6* on the liquid medium) was consistent with the previous hierarchical clustering analysis observed on the heatmap (Figure 3). With the PCA loadings, we were able to highlight some features of interest, namely, some specific peaks designated by their *m*/*z* value and their corresponding *t*_R_, which could be attributed to the clustering between the samples (Figure S1). Then, a supervised statistical study was performed, i.e., partial least square-discriminant analysis (PLS-DA), to highlight additional features of interest among the features with very important projection values (VIP) (Supplementary Figure S3). These were added to a list of important features that were clearly related to the culture conditions. In summary, all the features highlighted by means of univariate and multivariate analyses were combined and further studied (Table S1).

### 3.2. Identification of Features of Interest

In addition to the global metabolome comparison according to the culture conditions, features corresponding to the same compound (pseudomolecular, adduct, fragment ions), with the same *t*_R_ and displaying similar typical peak shapes in the extracted ion chromatograms (EICs) were combined. The monoisotopic mass of the corresponding compound was deduced, allowing us to suggest molecular formulae and allowing putative annotations based on the Dictionary of Natural Products (DNP) database (Table S1). When hits were found, their MS/MS spectra were compared, i.e., observed vs. reported, or directly compared to standards when available (isolated or commercial). In fact, only sterigmatocystin could be identified in a straightforward manner with absolute certainty as ST was previously isolated from the strain in the laboratory and used as a standard [50]. Its dihydro- derivative could also be identified as it presented a very similar fragmentation pattern to sterigmatocystin with an *m*/*z* difference of 2.0141 (Figure S4). To complete this study, a specific search on compounds already reported from the *Podospora* genus was also conducted by analyzing the *m*/*z* of the most common possible ions ([M+H]^+^, [M+Na]^+^, [M+K]^+^, [2M+H]^+^, and [2M+Na]^+^) in the data matrix. Finally, 57 different compounds were highlighted out of the features of interest. Most of them were found to be produced differently by *P. anserina*, regarding the culture conditions. While 40 of them presented hits in the database consulted as previously isolated from ascomycetes, including 8 already reported from *Podospora*, the remaining 26 were found to be potentially new (Table S1).

**Figure 4:**
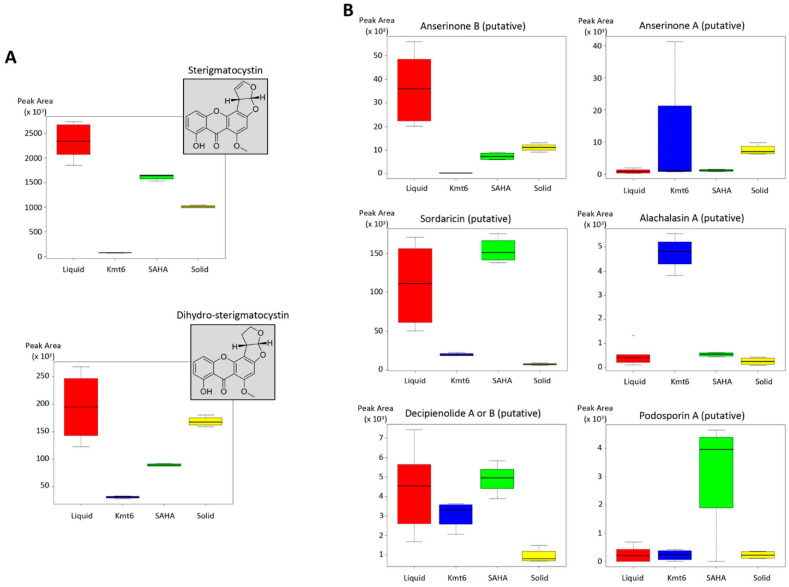
Boxplots representing the respective peak areas on the four different conditions for identified compounds sterigmatocystin (ST) (*t*_R_ = 10.21 min, *m*/*z* = 325.0705) and dihydro-sterigmatocystin (*t*_R_ = 9.84 min, *m*/*z* = 327.0846) (**A**) and for putatively identified compounds already reported in *Podospora* genus (**B**): Anserinone B (*t*_R_ = 5.97 min, *m*/*z* = 211.1032), anserinone A (*t*_R_ = 6.81 min, *m*/*z* = 231.0633), sordaricin (*t*_R_ = 9.50 min, *m*/*z* = 333.2032), alachalasin A (*t*_R_ = 9.59 min, *m*/*z* = 390.2196), decipienolide A or B (*t*_R_ = 10.64 min, *m*/*z* = 379.2052), and podosporin A (*t*_R_ = 12.67 min, *m*/*z* = 441.2586). Peak areas were obtained via integration of the corresponding peak on extracted ion chromatograms from UPLC data. “Liquid”: M2 liquid medium inoculated with WT; “Solid”: M2 solid medium inoculated with WT; “SAHA”: M2 liquid medium supplemented with 100 μM SAHA and inoculated with WT; “Kmt6”: M2 liquid medium inoculated with the *Kmt6* mutant strain. All the experiments were performed in quadruplicates, except for the condition “Liquid” for which 8 replicates were performed.

**Figure 5:**
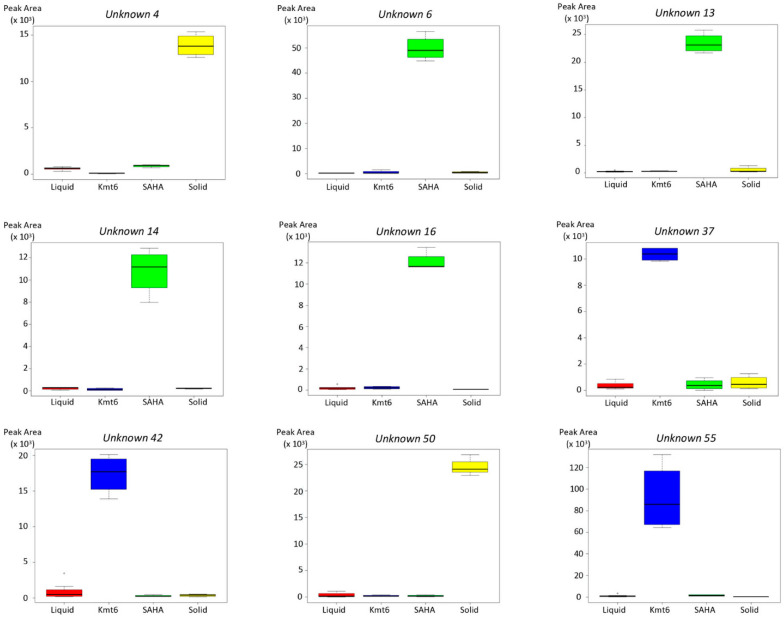
Boxplots representing the respective peak areas on the four different conditions for 9 selected unknown compounds, for which no match with compounds already reported from ascomycetes in DNP was obtained (from Table S1): Unknown 4 (*t*_R_ = 4.67 min, *m*/*z* = 203.0391), unknown 6 (*t*_R_ = 5.36 min, *m*/*z* = 243.1143), unknown 13 (*t*_R_ = 6.61 min, *m*/*z* = 263.1763), unknown 14 (*t*_R_ = 6.64 min, *m*/*z* = 424.1998), unknown 16 (*t*_R_ = 6.74 min, *m*/*z* = 222.1537), unknown 37 (*t*_R_ = 8.68 min, *m*/*z* = 469.164), unknown 42 (*t*_R_ = 10.17 min, *m*/*z* = 165.9889), unknown 50 (*t*_R_ = 11.24 min, *m*/*z* = 563.3802), and unknown 55 (*t*_R_ = 14.35 min, *m*/*z* = 428.3066). Peak areas were obtained by integration of the corresponding peak on extracted ion chromatograms from UPLC data. “Liquid”: M2 liquid medium inoculated with WT; “Solid”: M2 solid medium inoculated with WT; “SAHA”: M2 liquid medium supplemented with 100 μM SAHA and inoculated with WT; “Kmt6”: M2 liquid medium inoculated with the *Kmt6* mutant strain. All the experiments were performed in quadruplicates, except for the condition “Liquid” for which 8 replicates were performed.

Among compounds already reported from *Podospora*, ST belongs to the aflatoxin group and is known to be one of the major compounds produced by the *P. anserina* WT strain [50]. In this study, a comparison of the peak areas from the LC-UV analysis evidenced its higher production in the liquid culture medium when compared to other conditions (Figure 4A). Dihydrosterigmatocystin was found to be produced in similar amounts in liquid and solid mediums. In addition, other SMs already described in the *Podospora* genus, namely anserinone A and B, sordaricin, alachalasin A, decipienolide A or B, and podosporin A [48,59,60,61] were also putatively identified in this study (Figure 4B). Here again, as for ST production, the liquid medium appears to be the most accurate condition for the efficient production of anserinone B, sordaricin, and decipienolide A or B (Figure 4B). We can say here that, for ST, anserinone B, and decipienolide, the variation of medium states allowed us to easily find one culture condition that fits well with the high-level production of these metabolites. In contrast, in our conditions, the only way to trigger the production of alachalasin A and podosporin A seems to be chromatin modification by genetic deletion of *Kmt6* and the addition of the chemical epigenetic stimulant SAHA, respectively. Approaches involving the deregulation of chromatin are then very complementary to variations of culture conditions. In all the cases, the solid medium, which is not usual for *P*. *anserina* cultivation, does not seem very suitable for the production of metabolites previously identified. However, cultivation under this condition gave many specific unidentified compounds, as shown on the heatmap (Figure S1 and Table S1). In addition, when combined, the four conditions investigated in this study revealed an uninvestigated secondary metabolism from this strain that could not possibly be detected on usual liquid cultivation, highlighting potentially new compounds of interest for different purposes (such as therapeutics) (Figure 5). Further studies should be conducted to isolate and characterize these compounds.

## 4. Discussion

Despite the fact that the biosynthetic potential of filamentous fungi has been usually recognized at the genome level, the efficient production of metabolites is often hampered by the silent expression of most BGCs under laboratory conditions [3]. To counteract this thorny problem, some smart strategies have been developed in the past decade, based on targeted or global approaches. In previous work, we showed that targeted modification techniques could lead to the functional characterization of ST, a predominant metabolite in the filamentous fungal model *P. anserina* in liquid culture [50,51], but such an approach obviously limits access to larger metabolite exploitation. In this work, our main objective was to compare the global strategies usually used to deregulate SM gene clusters and illustrate the importance of complementary strategies as a foundation to activate silent BGCs and discover specific products of interest in the model fungus *P. anserina*. At this stage, we did not aim to identify new compounds, which can be performed in future works, but rather to rely on SMs already identified (or putative) in the databases to compare various approaches.

In our work, the most striking differences in metabolic profiles definitely occur between two very simple culture conditions: A liquid medium vs. a solid medium, which does not appear to be such a drastic change in culture conditions. In fact, previous studies revealed that a variety of culture conditions, e.g., solid vs. liquid, shaken vs. still, and high-density vs. low-density, have an extensive influence on microbial physiology [62,63,64,65]. In *Aspergillus niger*, the fungal transcriptomic response to sugar beet pulp was significantly different between solid culture and submerged culture, the latter inducing wider transcriptome variability [66]. Additionally, the fungal proteome and secretome complexity were affected by the cultivation modes in *Aspergillus* and *Trichoderma* species [67,68,69]. In *Penicillium ubiquetum* isolated from the blue mussel *Mytilus edulis*, it has been shown that, following the OSMAC approach, the culture performed on the seawater CYA (Czapek Yeast extract Agar) medium selectively enhances the production of some structurally related compounds [70]. Apart from the obvious differences in gene expression and enzyme production, the actual production of bioactive metabolites in filamentous fungi was also highly reliant on culture conditions [71]. Submerged cultivation was considered the preferred option for fungal fermentation and metabolite extraction in the laboratory and industry, which allows for the maintenance of stable ambient conditions, the standardization of biotechnological procedures, and the continuous depletion of dissolved substrates [72,73]. Nevertheless, it has been proven that the solid culture was better than the liquid culture for the production of valuable metabolites in *Fusarium* and *Penicillium* species [62,74,75]. In particular, some fungal antibiotics were only generated under solid conditions [76]. In *Fusarium graminearum* and *Fusarium solani*, almost identical SM profiles in the agar plate and glass bead cultures can be detected, whereas lower yields of SMs were obtained on the liquid culture, as exemplified by the absence of major products (zearalenone and aurofusarin) in this case [75]. In *Aspergillus terreus*, it has been shown that the yield of lovastatin decreased by 50% in submerged cultures [77]. With regard to *P. anserina*, solid-state cultivation seems to be a noteworthy strategy because the corresponding metabolomic profile is completely different from liquid culture to solid culture. In this study, we showed that the highest proportion of significant unique features was observed on solid medium, whatever the threshold used. It has been suggested that, to some extent, the solid culture medium is somewhat closer to the environment for many microorganisms [66,78], thus allowing the expression of a broad range of BGCs useful in the natural environment. Indeed, fungal hyphae in solid culture are likely exposed to variable nutritional substrates and environmental factors during the extension process, which likely mimic their natural habitat. In contrast, mycelium handles a priori more uniform substrates in liquid culture [79,80,81]. We noted that the preferred habitat for *P. anserina* is a solid substrate with less moisture content in nature, i.e., herbivore dung [44]. Therefore, one could expect that the cultivation of *P. anserina* in solid media might be more suitable for the induction of cryptic SMs, which are not expressed by conventional fermentation in liquid culture.

Beyond culture conditions, which can be easily modified but whose possibilities are almost unlimited, we also propose global approaches based on the deregulation of chromatin, as its related structure has been proven to be closely associated with the regulation of SMs in fungi [3,82]. It is known that histone H3 methyltransferases and histone deacetylases can convert chromatin from an active euchromatic state to a silent heterochromatic state [43]. To suppress this process, Δ*Kmt6* and SAHA-treated wild-type strains were used in our study, which might thereby allow the activation of metabolic pathways through inhibiting histone methylation and deacetylation in *P. anserina*. Notably, the deletion of *Kmt6* in *P. anserina* showed drastic physiological defects in vegetative growth and sexual development [53]. Such approaches based on chromatin deregulation have already been successfully applied to other fungi. The conserved histone methyltransferase LaeA, acetyltransferase HatA, demethylase HdmA, and deacetylase HdaA have been reported to regulate primary or secondary metabolism in *A. nidulans*, *F. fujikuroi*, *Magnaporthe oryzae,* and *Ustilago maydis* [83,84,85,86]. Furthermore, deletion of the H3K27me3 methyltransferase Kmt6 can result in the activation of hundreds of SM biosynthesis-associated genes and the induction of cryptic BGCs, which has been investigated in *F. fujikuroi*, *F. graminearum*, *Epichloë festucae,* and *Ustilaginoidea virens* [32,87,88,89,90]. Applying inhibitors of histone-modifying enzymes is equally effective for metabolite modulation in fungi. Among the chemical histone deacetylase inhibitors, SAHA is widely used as an effective approach to identify novel natural products in fungi, especially for those non-model fungi that are not genetically tractable [29]. For instance, the addition of SAHA in a liquid medium induced the production of eight metabolites in the endophytic fungus *Botryosphaeria mamani* [91]. Previous studies showed that SAHA treatment on various fungi caused the misregulation on gene expression and the perturbation of metabolism. Therefore, SAHA has been well applied to activate the silent metabolic pathways and enhance the yield of native products in fungi [29,82].

Overall, in this study, we found that the deletion of *Kmt6* or the addition of SAHA in *P. anserina* liquid culture led to significantly different metabolic profiles, both of which neither overlapped with those in the solid culture nor in the liquid culture. Our results indicated that a batch of SMs is exclusively detected under unique conditions and further implied the large untapped capacity of metabolite production in *P. anserina*.

The present study demonstrated a distinct but complementary metabolic profile when treated with different culture conditions and modified epigenetic regulation. We showed that, individually, each mean used to deregulate the silent BGCs impacts the SM profile in its own way and that a high level of specific/unique features overexpression is observed. Then, some well-known compounds in *P. anserina* such as ST and anserinone B were specifically found in the liquid medium, which is the commonly used culture condition for this fungus. Some other unknown compounds are specifically found in other conditions that remain to be explored in future works, such as compounds 4 and 50 (WT in a solid medium), compounds 6, 13, 14, and 16 (WT in a liquid medium + SAHA), and compounds 37, 42, and 55 (Δ*Kmt6* strain in a liquid medium) (Figure 4 and Figure 5). It is thus clear that the different combined approaches used to deregulate the silent BGCs and based on the metabolomics approach are very promising to isolate compounds not yet identified in databases and therefore potentially new.

## Data Availability

The data presented in this study are included in the main text and Supplementary Materials.

## Supplementary Materials

The following are available online at https://www.mdpi.com/article/10.3390/jof9010009/s1, Table S1: List of highlighted features of interest from *P. anserina* cultures and dereplication according to a search in the Dictionary of Natural Products; Figure S1: Multivariate study of the *P. anserina* WT strain in relation to four different conditions; Figure S2: Overview of the principal component analysis of the normalized and Pareto-scaled matrix; Figure S3: Score plot and VIP features; Figure S4: UPLC chromatograms.
