# Supplementary material for: Complementary Strategies to Unlock Biosynthesis Gene Clusters Encoding Secondary Metabolites in the Filamentous Fungus Podospora anserina
Source: J Fungi (Basel). 2022 Dec 21;9(1):9. doi: 10.3390/jof9010009 (PMC9864250; doi:10.3390/jof9010009)
Supplement: Supplementary file 1 [file jof-09-00009-s001.zip › jof-2052934-supplementary.pdf]

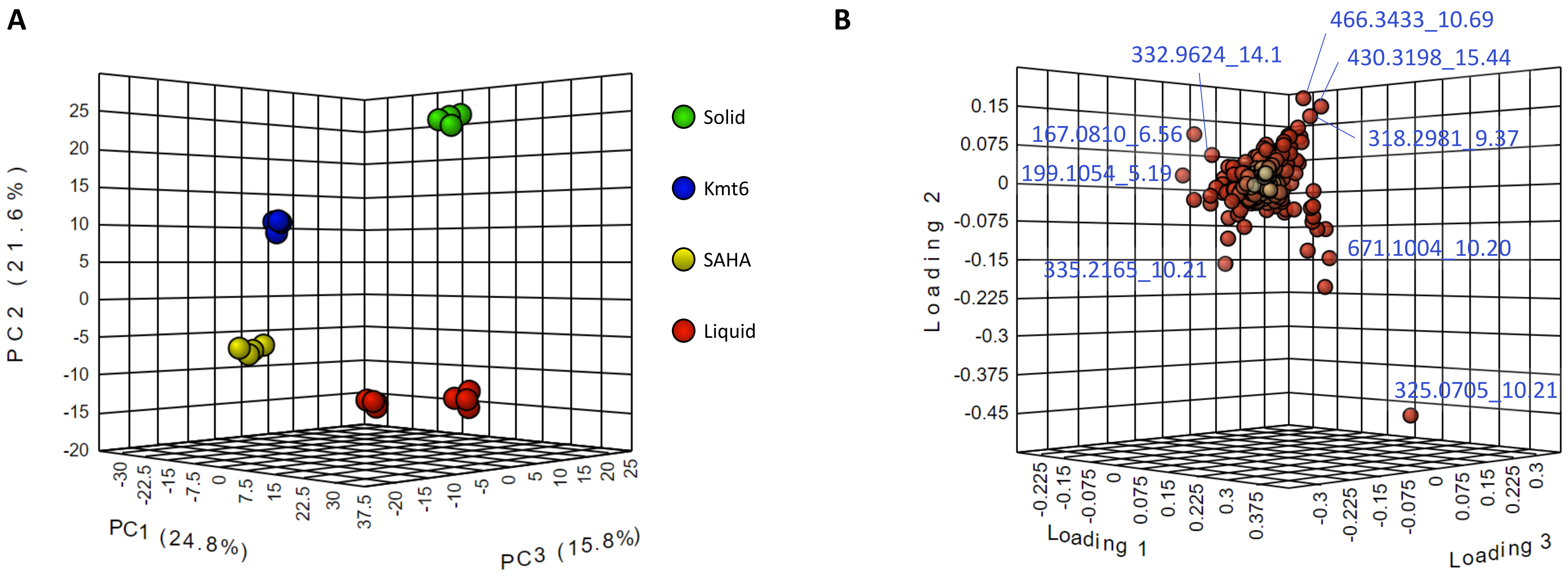

Figure S1. Multivariate study of the *P. anserina* WT strain in relation to four different conditions. “Liquid”: M2 liquid medium inoculated with WT; “Solid”: M2 solid medium inoculated with WT; “SAHA”: M2 liquid medium supplemented with 100 µM SAHA and inoculated with WT; “Kmt6”: M2 liquid medium inoculated with the Kmt6 mutant strain. Principal component analysis (PCA) 3D-score plot (**A**) figuring PC1 vs PC2 vs PC3 on the complete data set (Pareto scaling) and loadings plot (**B**) representing the variability of the samples (with 24.8% of variance explained on PC1 and 21.6% on PC2) and the weight of the different variables, respectively. Some features with important coefficient in the PCA are shown in blue. All the experiments were performed in quadruplicates, except for the condition “Liquid” for which 8 replicates were done.

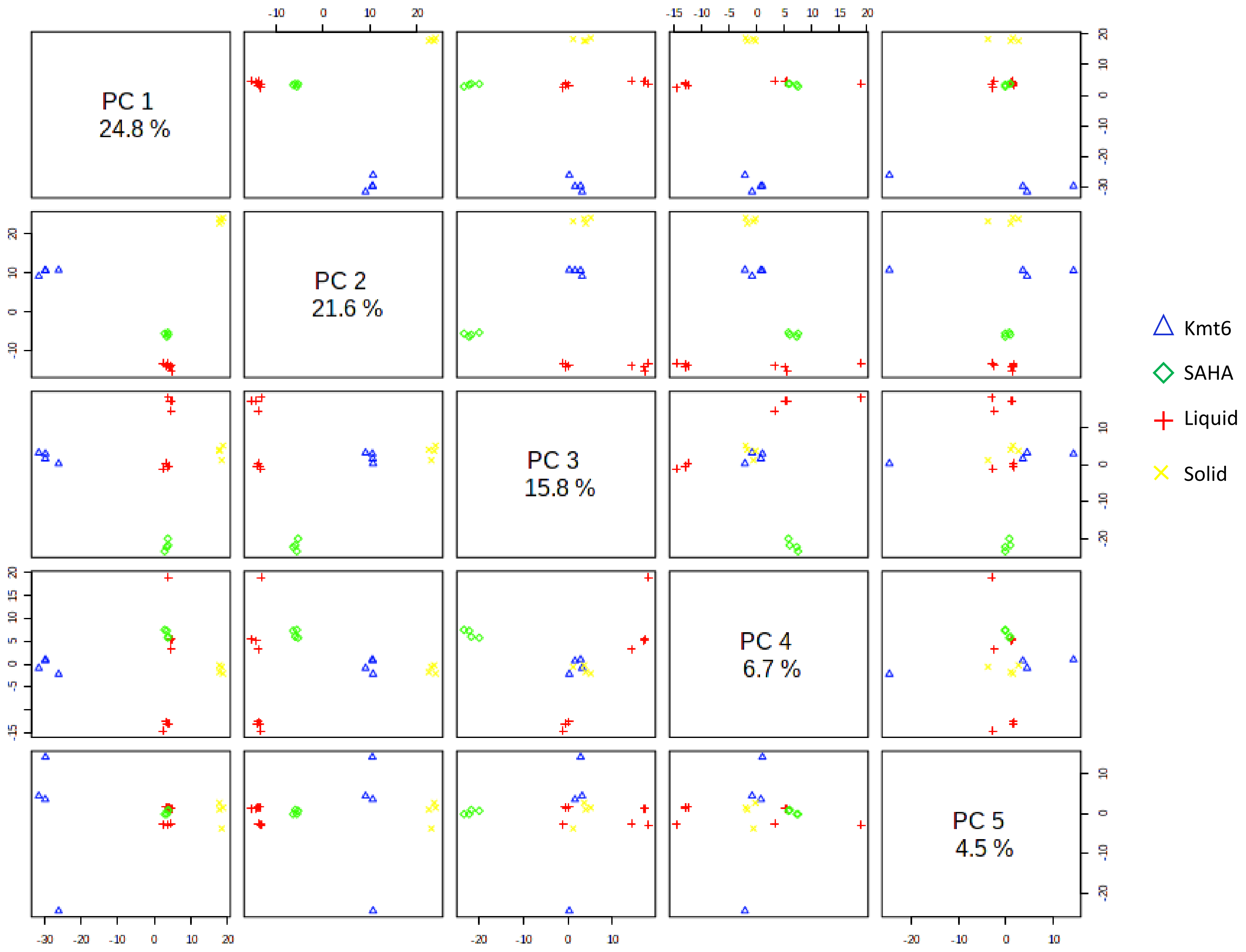

Figure S2. Overview of the principal component analysis of the normalised and Pareto-scaled matrix illustrating the 5 first principal components, which account for 73.6% of total variance.

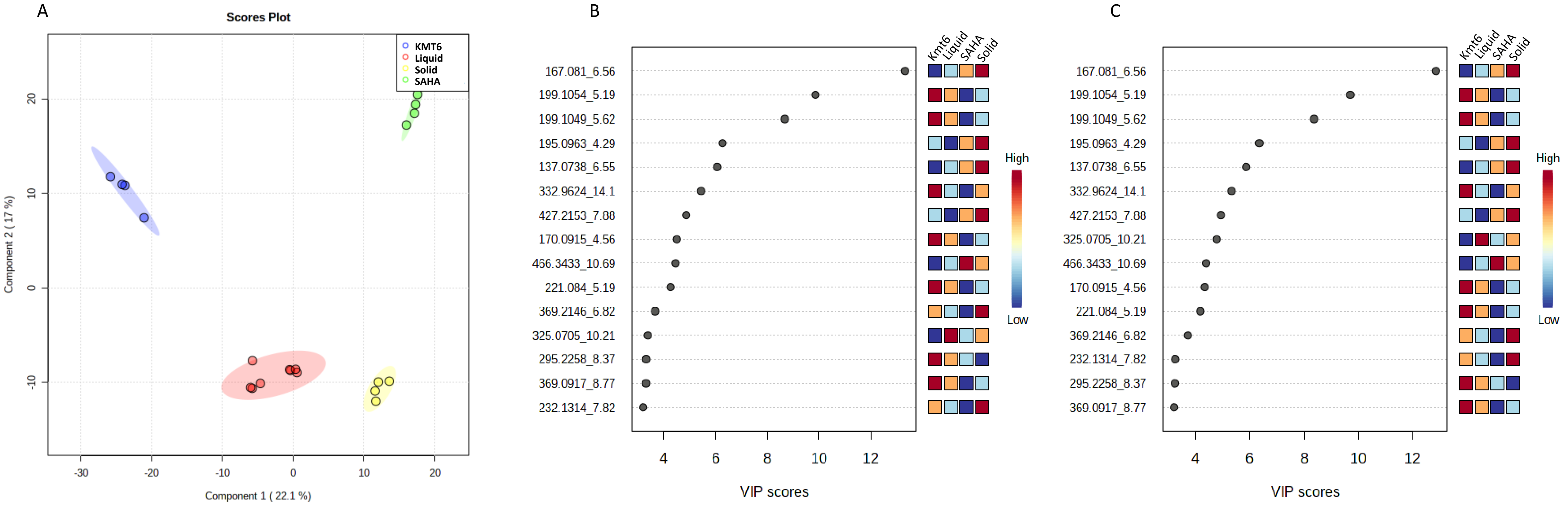

Figure S3. (**A**) Score plot for PLS-DA of the normalised and Pareto-scaled matrix; (**B**) VIP features for component 1 of PLS-DA; (**C**) VIP features for component 2 of PLS-DA.

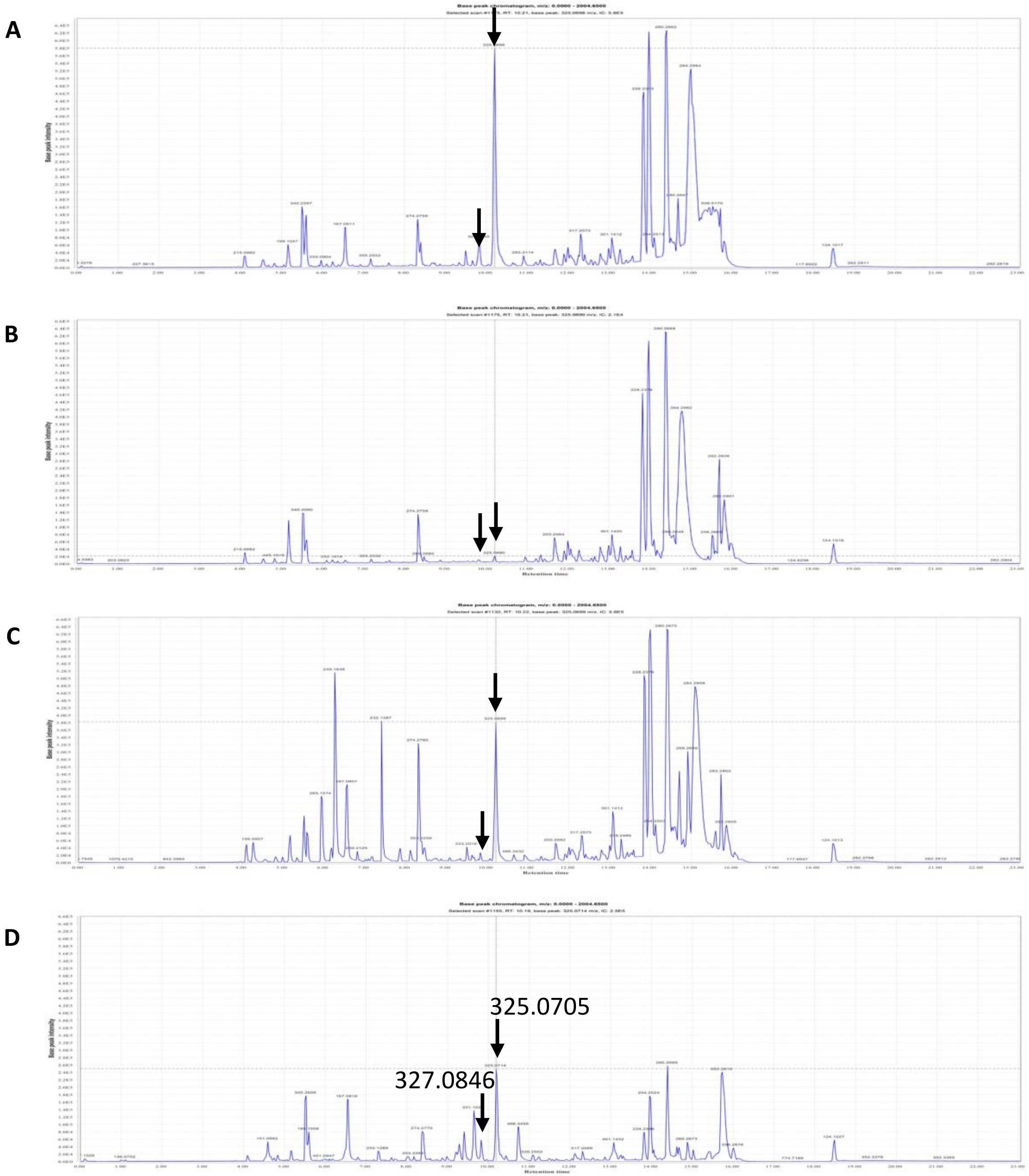

Figure S4. HPLC chromatograms showing the identified compounds sterigmatocystin (ST) (t_R = 10.21 min, *m/z* = 325.0705) and dihydro-sterigmatocystin (t_R = 9.84 min, *m/z* = 327.0846) under different conditions (**A**) M2 liquid medium inoculated with WT; (**B**) M2 liquid medium inoculated with the Kmt6 mutant strain; (**C**) M2 liquid medium supplemented with 100 μM SAHA and inoculated with WT; (D) M2 solid medium inoculated with WT.

Table S1

Cpd	Description				Hits in DNP (Ascomycetes)	Hits in NP Atlas (Ascomycetes)	Hits in SciFinder ("Purification or recovery" or "Natural product occurrence")	New compound 1: high chance 0: low chance	Hits in databases (bold corresponds to compounds already described in Ascomycetes red to compounds already described in Sordariales * to compounds already described in *Podospora*)	Variation observed (average peak areas)				Statistical analyses	
	T_R	*m/z*	Ion species	Monoisotopic mass						WT-Liquid	Kmt6-Liquid	WT-SAHA	WT-Solid	Multivariate	Univariate
1	4.11	215.0984	$[M+H]^+$	214.0911	8 (0)	1 (1)		0	**soboliferamide; cyclo(aspartylvalyl)**	→ 116833	↓ 81169	↑ 178820	↓ 40255	✓	✓
2	4.29	195.0963	$[M+H]^+$	194.0890	173 (38)	48 (37)		0	**kobifuranone B; angupyrone B; aspergone A-B; pestalafuranone A-B,D,J** ; 2-amino-2-deoxyglucose-oxime; ...	↓ 511	↓ 290	↑ 305264	↓ 1274	✓	✓
3	4.56	170.0915	$[M+H]^+$	169.0842	11 (0)	0	5103 (13)	0	methylhistidine (**anserine derivative**)	↑ 105707	↓ 45979	↓ 43416	↓ 34724	✓	✓
4	4.67	203.0391	$[M+Na]^+$	180.052	9 (0)	0	2322 (1)	1	3-acetyl-2,5-diamino-1,4-benzoquinone; makaluvic acid C; …	↓ 605	↓ 94	↓ 905	↑ 13897		✓
5	5.19	199.1054	$[M+H]^+$	198.0981	9 (3)	2 (0)		0	**xestostreptin; cyclo(dehydroalanyl-N,O-dimethylthreonyl); cyclo(prolylthreonyl); ...**	↑ 273172	↑ 336740	↑ 294192	↑ 89387	✓	✓
6	5.36	243.1143	$[M+Na]^+$	220.1280	17 (1)	1 (1)		0	**collacyclumine C**	↓ 206	↓ 471	↑ 49864	↓ 440		✓
7	5.55	176.0836	$[M-H_2O+H]^+$	193.0824	0	0	19 (0)	1		↓ 316	↓ 371	↑ 46635	↓ 246		✓
8	5.61	199.1049	$[M+H]^+$	198.0976	9 (3)	2 (0)		0	xestostreptin; cyclo(dehydroalanyl-N,O-dimethylthreonyl); cyclo(prolylthreonyl); ...	↑ 449423	↓ 228061	→ 308169	↓ 186616	✓	✓
9	5.97	211.1029	$[M+H]^+$	210.0956	196 (46)	49 (33)		0	**anserinone B***	↑ 36271	↓ 382	↓ 7397	↓ 11147	✓	✓
10	5.98	255.0897	$[M+H]^+$	254.0808	57 (11)	15 (11)		0	**clearanol J; …**	↑ 101671.6	↓ 296.8	↓ 4762.1	↓ 15037.4	✓	
11	5.98	401.0915	$[M+H]^+$	400.0842	15 (4)	5 (4)		0	**cercophorin A; griseucin C; paeciloquinone B; 3'-acetylversiconal; methylstictic acid; diffractione A**	↓ 725	↓ 766	↓ 1048	↑ 46624	✓	
12	6.56	167.0810	$[M+H]^+$	166.0737	25 (2)	0		0	**cephalosporidine ; 5H-Pyrrolo[3,4-d]piperidine-2-carboxylic acid**	↓ 231603	↓ 29817	↑ 1042055	→ 699533	✓	✓
13	6.61	263.1763	$[M+H]^+$	262.1720	35 (0)	2 (0)	68208 (72)	1	N1-acetyl-N7-phenylacetyl cadaverine; bohemamine G; (+)-oxysophocarpine; 2,3,5-trihydroxytridecanoic acid; octyl β-D-xylopyranoside	↓ 279	↓ 295	↑ 23447	↓ 537		✓
14	6.64	424.1998	$[M+H]^+$	423.1912	7 (0)	0	8113 (32)	1	hodgsonine; tripterregeline A; florosenine; …	↓ 224	↓ 129	↑ 10793	↓ 221		✓
15	6.71	461.1803	$[M+H]^+$	460.173	39 (2)	2 (2)		0	**daldinin E; daldinin F**	↓ 330	↓ 231	↑ 26037	↓ 303		✓
16	6.74	222.1537	$[M+H]^+$	221.1464	37 (0)	2 (0)	98258 (106)	1	roripamine; YUA001; TMC-49A; alchorneine; isoalchorneine; …	↓ 204	↓ 230	↑ 12096	↓ 90		✓
17	6.81	231.0633	$[M+Na]^+$	208.0741	186 (48)	46 (33)		0	**anserinone A***	↓ 956	↑ 11078	↓ 1249	→ 7552		
18	6.81	209.0903	$[M+H]^+$	208.083	20 (1)	2 (0)		0	**2-[(2-Hydroxypropanoyl)amino]benzamide**	↓ 2317	↓ 248	↓ 3971	↑ 173048	✓	
19	6.82	369.2146	$[M+H]^+$?	368.2073	43 (3)	2 (2)		0	**mollenine A ; PJ 147; PJ 157**	↓ 413	↓ 303	↑ 98958	↓ 241	✓	✓
			$[M+Na]^+$?	346.2254	7 (0)	1 (0)			nevaltophin A; *O*-acetyl-serratinidine; …						
			$[M-H_2O+H]^+$?	386.2168	6 (0)	1 (0)			avaroferin; anomaline						
20	6.92	248.1328	$[M+H]^+$	247.1255	12 (1)	1 (1)		0	**(±)-isoalternatine A**	↓ 193	↓ 183	↑ 29191	↓ 288		✓
21	7.11	426.2193	$[M+H]^+$	425.2202	3 (2)	2 (1)		0	***N*-(2-Hydroxyethyl)monascorubramine; circinatin**	↓ 263	↓ 107	↑ 27248	↓ 206		✓
22	7.17	355.2552	$[M+H]^+$?	354.2479	1 (0)	0	9990 (3)	1	N2-(9Z-Tetradecenoyl)-glutamine; …	→ 52134	→ 45746	↑ 72050	↓ 16012	✓	✓
			$[M+Na]^+$?	337.2214	3 (1)	0	1913 (13)		**antibiotic Ro 09-1547; 4-Me ether, 3-O-(aminoacetyl)**; 1-epi-17S-hydroxymalycorin A; wardianumine A						
			$[M-H_2O+H]^+$?	332.2660	39 (0)	0	1722 (55)		ankoline; belamcandol B; garcinielliptone N; …						
23	7.20	490.2253	$[M+Na]^+$	467.2161	1 (0)	0	8772 (19)	1	*N*-tri-Ac-sorbistin D	↓ 357	↓ 92	↑ 42816	↓ 159	✓	✓
24	7.28	252.1272	[M+H]+	251.1157	42 (6)	14 (10)		0	**cladosin E; cladosporiomin E; huaspenone C; paraphaeosphaeride B; rigidiusculamide A; BSF-A; …**	↓ 384	↓ 246	↓ 478	↑ 93458	✓	✓
25	7.41	395.1905	$[M+H]^+$	394.1832	40 (1)	3 (3)		0	**acremine G; staprexanthone A; penicitrinone F**	↑ 81108	↓ 71	↓ 143	↓ 1423	✓	
26	7.41	252.0910	$[M+H]^+$	251.0837	15 (2)	5 (4)		0	**prolipyrone C; hypoxyloamide; phomarosine A; pyranonigrin F**	↓ 482	↓ 244	↓ 547	↑ 12107		✓
27	7.71	351.2497	$[M-H_2O+H]^+$	368.2509	70 (2)	6 (0)		0	**betaenone B; pertusarinic acid; …**	↓ 746	↓ 353	↓ 925	↑ 6296		✓
28	7.86	427.2153	$[M+H]^+$	426.2080	71 (18)	12 (12)		0	**ardeemin; andibenin B; brasilianoid L; chrodrimanin E; cochliobolic acid; dicitrinol A; dicitrinol B; diaporindene A-D; emericellic acid; emeridone D; hesseltin C-D; insuetolide C; isoaustinone; lenormandin E; neoaustin;** MP-6	↓ 160	↓ 189	↑ 180421	↓ 650	✓	✓
29	8.14	427.2141	$[M+H]^+$	426.2080	71 (18)	12 (12)		0	**ardeemin; andibenin B; brasilianoid L; chrodrimanin E; cochliobolic acid; dicitrinol A; dicitrinol B; diaporindene A-D; emericellic acid; emeridone D; hesseltin C-D; insuetolide C; isoaustinone; lenormandin E; neoaustin;** MP-6	↓ 376	↓ 438	↑ 24263	↓ 285		✓
30	8.27	371.1062	$[M+H]^+$?	370.0989	158 (12)	16 (9)		0	**chaetoxanthone A; didemethylasterriquinone D; cladosporol E; almarumycin C16; norsolorinic acid; 5'-epi-averufanin; 2-O-methylbutyrolactone II; asperphenol C;** spirobrocazine A; eutypellazine P; ...	→ 14136	↑ 21124	↓ 5283	↓ 6419	✓	✓
			$[M+Na]^+$?	348.1170	299 (13)	21 (13)			**15-*O*-desmethyl-5Z-7-oxozeaenol; 5'-hydroxymonocillin III; roseopurpurin C-D; paraphaeosphaerin C; colletotrichone A; mycousfuran A-B; auranomide A; auranomide C;** oliverin…						
			$[M-H_2O+H]^+$?	388.1084	0	0									
31	8.36	253.1378	$[M+H]^+$?	252.1305	130 (32)	41 (22)		0	**chaetolactone; aspergillide C; frequentin; dendrodochol A; phomoxide; pestalotioprolide E-G; …**	↓ 853	↓ 924	↑ 18169	↓ 653		✓
			$[M+Na]^+$?	230.1486	70 (13)	21 (9)			**cladospolide F; talaromycin A; graphostromol K; cladospolide F; cladosporacid A-C; noraspiperidine; …**						
			$[M-H_2O+H]^+$?	270.1400	47 (16)	19 (13)			**clavicipitic acid ; molliclavine; quinolactacin A; quinolactacin E; xylaric acid A; ficipyrone A; botrylacone; dendrodochol D; peniisocoumarin A; cladosin L; …**						
32	8.36	213.1568	$[M+H]^+$	212.1495	10 (2)	3 (1)			**cyclo(leucylvalyl); cyclo(isoleucylvalyl)**	→ 95606	↓ 30555	→ 104219	↑ 191048		✓
33	8.36	699.4177	$[2M+H]^+$	349.2052	8 (1)	2 (1)		0	**lecanindole A**	→ 52217	↓ 13303	→ 51815	↑ 113167	✓	✓
34	8.36	353.2283	$[M+Na]^+$	330.2391	45 (4)	3 (3)			**penicitide B; mollisinol B; 9,10,11-trihydroxy-12-octadecenoic acid**	→ 143689	↓ 60890	→ 153222	↑ 220396	✓	✓
35	8.47	477.2637	$[M+H]^+$?	476.2564	33 (0)	2 (1)		0	**penitanzchroman**	↓ 467	↑ 36284	↓ 218	↓ 189	✓	✓
			$[M+Na]^+$?	454.2745	120 (0)	5 (1)			**(7S,11S,12S,14R)-14-methoxyamentol quinone**						
			$[M-H_2O+H]^+$?	494.2659	63 (1)	4 (0)			**communesin C**						
36	8.62	700.3366	$[M+H]^+$	699.3293	4 (0)	0	383 (6)	1	L-histidyl-L-histidylglycyl-L-histidyl-L-arginylglycine; euphocharacin D; euphodendrophane A; nicaeenin G…	↓ 156	↓ 295	↑ 28254	↓ 161		✓
37	8.66	469.164	$[M+H]^+$?	468.1566	5 (0)	0	1306 (7)	1	dichamanetin; isoriccardinquinone A; marchantin N; …	↓ 344	↑ 10373	↓ 423	↓ 575		✓
			$[M+Na]^+$?	446.1748	7 (1)	1 (1)	2555 (18)		**oxysporizoline;** achlisocoumarin IV; cudracuspiflavanone A; trigoxyphin P; …						
			$[M-H_2O+H]^+$?	486.1662	18 (0)	1 (0)	1996 (31)		fasamycin H; vitisinol E; viniferether A; mersiphylline A; kopsimaline A; kopsimaline F; ...						
38	9.28	286.1432	$[M+H]^+$?	285.1359	59 (1)	4 (2)		0	**N-carbobenzoxy-L-phenylalanilol; piperine**	↓ 664	↓ 520	↓ 894	↑ 64672	✓	✓
			$[M+Na]^+$?	263.1540	22 (1)	5 (3)			**pyridoxatin; tolypyridone D; preussin C**						
			$[M-H_2O+H]^+$?	303.1454	46 (2)	4 (2)			**solanapyrone E-F; banchromene**						
39	9.37	318.2981	$[M+H]^+$	317.2908	18 (0)	1 (0)	97 (4)	1	4-*N*-hydroxy-1-amino-16-guanidino-4,8,12-triazahexadecane; halisphingosine B; phytosphingosine; 4-hydroxysphinganine; …	↓ 41844	↓ 6445	↓ 50143	↑ 263187	✓	✓
40	9.5	333.2032	$[M+H]^+$?	332.1959	1053 (24)	40 (18)		0	**sordaricin***	↑ 109665	↓ 19689	↑ 153837	↓ 7390	✓	✓
41	9.84	327.0846	$[M+H]^+$	326.0773	98 (6)	8 (4)		0	**dihydrosterigmatocystin***	↑ 195163.0	↓ 31221.7	→ 89669.8	↑ 168608.6	✓	✓
42	10.17	165.9889	$[M+H]^+$	164.9816	0	0	3 (0)	1		↓ 944.2	↑ 17342.8	↓ 295.8	↓ 377.3	✓	✓
43	10.20	325.0705	$[M+H]^+$	324.0632	29 (7)	9 (3)		0	**sterigmatocystin***	↑ 2346136	↓ 71245	↑ 1618388	→ 1008674	✓	✓
44	10.20	464.3257	$[M+H]^+$?	463.3184	0	0	717 (5)	1		↓ 2387	↓ 397	↓ 9814	↑ 64846	✓	✓
			$[M+Na]^+$?	441.3365	1 (0)	0	412 (2)		pyrinodemin E						
			$[M-H_2O+H]^+$?	481.3279	0	0	100 (0)								
45	10.43	464.3287	$[M+H]^+$?	463.3214	0	0	717 (5)	1		↓ 523	↓ 259	↓ 1201	↑ 91129	✓	✓
			$[M+Na]^+$?	441.3398	1 (0)	0	412 (2)		pyrinodemin E						
			$[M-H_2O+H]^+$?	481.3312	0	0	100 (0)								
46	10.69	466.3433	$[M+H]^+$?	465.3360	4 (0)	3 (0)	440 (0)	1	olivoretin A-C; olivoretin E	↓ 30704	↓ 960	↓ 99936	↑ 372084	✓	✓
			$[M+Na]^+$?	443.3541	0	0	240 (2)								
			$[M-H_2O+H]^+$?	483.3455	1 (0)	0	343 (0)		blastmycetin D						
47	10.93	293.2106	$[M-H_2O+H]^+$	310.2128	70 (4)	15 (2)		0	**cephalosporolide H; graphostromol E; lachnellukoic acid; 12,13-epoxy-11-hydroxy-9,15-octadecadienoic acid**	↑ 69178	↓ 22938	↑ 84534	↓ 9889	✓	✓
48	11.09	671.4617	$[M+H]^+$	670.4544	0	0	179 (1)	1		↓ 344	↓ 508	↓ 485	↑ 12275		✓
49	11.22	739.4787	$[M+Na]^+$	716.4894	4 (0)	3 (0)	145 (4)	1	mycolactone G; spirangien B; spirangien M; spirangien P	↓ 337	↓ 171	↓ 329	↑ 19273		✓
50	11.24	563.3802	$[M+H]^+$	562.3729	10 (0)	0	540 (12)	1	buxanaldinine; buxandrine; acanthomanzamine D; …	↓ 355	↓ 231	↓ 195	↑ 24478		✓
51	11.97	405.2612	$[M+H]^+$	404.2540	72 (4)	10 (5)		0	**yanuthone M; lovastatin; 17-*O*-methyltricycloalternarene D; 19-succinoyl-16-kaurene-3,19-diol; berkchaetoazaphilone C; trichodermolide**	↓ 290	↓ 458	↑ 5336	↓ 178		✓
52	12.33	179.1526	$[M+H]^+$?	178.1453	8 (0)	4 (0)	8677 (28)	1	2,5-dimethyl-3-(2-methylbutyl)pyrazine; ...	↑ 72883	↓ 25947	↑ 84149	↓ 29882	✓	✓
			$[M-H_2OH]^+$?	196.1548	2 (0)	0	37868 (2)		bis(pentamethylene)urea; epiquinamide						
53	12.49	480.2132	$[M+H]^+$	479.2059	3 (2)	2 (2)		0	**notoamide O; notoamide H**	↓ 432	↑ 7109	↓ 247	↓ 397		✓
54	13.34	329.1648	$[M+Na]^+$	306.1841	72 (9)	18 (12)		0	**aspergiloid I; ML 236A; 3,5-dihydro-3-oxo-ML 236C; graminin B; tanzawaic acid H-J & O; deacylcompactin; scopararane G; …**	↓ 10402	↓ 1456	↓ 3990	↑ 120093	✓	✓
55	14.1	332.9624	$[M+H]^+$	331.9551	0	0	41 (1)	1	2,2'-benzothiazolyl disulfide	↓ 1120	↑ 92043	↓ 1602	↓ 455	✓	✓
56	15.44	430.3198	$[M+H]^+$?	429.3125	0	0	1142 (1)	1	methyl 2-deoxy-2-[[(9Z)-1-oxo-9-hexadecen-1-yl]amino]-β-D-glucopyranoside	↓ 35110	↓ 3606	↓ 27852	↑ 336262	✓	✓
			$[M+Na]^+$?	407.3308	0	0	360 (0)								
			$[M-H_2O+H]^+$?	447.3220	2 (0)	0	385 (3)		antibiotic TKR 459						
57	15.44	422.1906	$[M+H]^+$?	421.1833	12 (1)	1 (1)		0	**farinosone B**	↓ 812	↓ 136	↓ 190	↑ 18608		✓
			$[M+Na]^+$?	399.2014	12 (2)	3 (3)			**spirodihydrobenzofuranlactam V; chartarlactam A; (±)-childinin C**						
			$[M-H_2O+H]^+$?	439.1928	3 (0)	0									
